# Chemoinformatic Identification of Novel Inhibitors against *Mycobacterium tuberculosis* L-aspartate α-decarboxylase

**DOI:** 10.1371/journal.pone.0033521

**Published:** 2012-03-28

**Authors:** Reetu Sharma, Roopa Kothapalli, Antonius M. J. Van Dongen, Kunchithapadam Swaminathan

**Affiliations:** 1 Department of Biological Sciences, National University of Singapore, Singapore; 2 Department of Obstetrics and Gynaecology, National University of Singapore, Singapore; 3 Duke-NUS Graduate Medical School, National University of Singapore, Singapore; Bioinformatics Institute, Singapore

## Abstract

L-Aspartate α-decarboxylase (ADC) belongs to a class of pyruvoyl dependent enzymes and catalyzes the conversion of aspartate to β-alanine in the pantothenate pathway, which is critical for the growth of several micro-organisms, including *Mycobacterium tuberculosis* (Mtb). Its presence only in micro-organisms, fungi and plants and its absence in animals, particularly human, make it a promising drug target. We have followed a chemoinformatics-based approach to identify potential drug-like inhibitors against *Mycobacterium tuberculosis* L-aspartate α-decarboxylase (MtbADC). The structure-based high throughput virtual screening (HTVS) mode of the Glide program was used to screen 333,761 molecules of the Maybridge, National Cancer Institute (NCI) and Food and Drug Administration (FDA) approved drugs databases. Ligands were rejected if they cross-reacted with S-adenosylmethionine (SAM) decarboxylase, a human pyruvoyl dependent enzyme. The lead molecules were further analyzed for physicochemical and pharmacokinetic parameters, based on Lipinski's rule of five, and ADMET (absorption, distribution, metabolism, excretion and toxicity) properties. This analysis resulted in eight small potential drug-like inhibitors that are in agreement with the binding poses of the crystallographic ADC:fumarate and ADC:isoasparagine complex structures and whose backbone scaffolds seem to be suitable for further experimental studies in therapeutic development against tuberculosis.

## Introduction

L-Aspartate α-alpha decarboxylase (ADC, EC 4.1.1.11), encoded by the *panD* gene, is a lyase and catalyzes the decarboxylation of aspartate to β-alanine, which is essential for D-pantothenate formation (Fig. S1). Mutants of the *panD* gene are defective in β-alanine biosynthesis [1]. β-alanine and D-pantoate condense to form pantothenate, a precursor of coenzyme A (CoA), which functions as an acyl carrier in fatty acid metabolism and provides the 4′-phosphopantetheine prosthetic group in fatty acid biosynthesis, an essential need for the growth of several micro-organisms, including *Mycobacterium tuberculosis* (Mtb) [2], [3], the causative bacterial agent of tuberculosis (Tb) [4].

The distinctive lipid rich cell wall of Mtb is responsible for the unusually low permeability, virulence and resistance to therapeutic agents [5], [6]. At the heart of the fight against tuberculosis lies its cell wall, a multilayered structure adorned with a number of lipo-glycans that protect the bacterium in antimicrobial defense against environmental stresses and treatment. Consequently, the metabolism and biosynthesis of lipids and lipo-glycans play a pivotal role in the intracellular survival and persistence of Mtb. Any impediment in the pantothenate pathway will therefore affect the survival of the bacterium. As Mtb is notorious to develop resistance towards drugs, progress in the treatment of tuberculosis will require us to identify new targets in pathways critical for the sustenance of Mtb, and to develop new drugs selectively inhibiting these targets so as to minimize drug resistance and potential side effects [7], [8]. Since pantothenate is synthesized only in microorganisms, fungi and plants, but not in humans, the enzymes that are involved in this biosynthetic pathway qualify to be potential targets for antibacterial and antifungal agents [9]. The absence of this pathway in humans ensures that any inhibitor or drug against ADC would have low toxicity in patients. In particular, the chance of side effects in a long term treatment procedure will be minimal. Moreover, the presence of the ADC gene in only one copy in the Mtb genome further enhances its importance as a suitable drug target.

MtbADC is translated as an unprocessed proenzyme (π-protein). It undergoes autocatalyzed cleavage between Gly24 and Ser25, where the serine is modified to a pyruvoyl group, resulting in the formation of 2.8 kDa β-chain and 11 kDa α-chain containing the N-terminal pyruvoyl group. This processed α form is necessary for the conversion of aspartate to β-alanine [10] and the mutation S25A makes the protein uncleavable and inactive [11]. So far, crystal structures have been determined for unprocessed (uncleaved) ADC from *E. coli* (PDB id: 1PPY) [12], Mtb (2C45) [13], and processed ADC from *E. coli* (1AW8) [14], *Francisella tularensis* (3OUG), *Campylobacter jejuni* (3PLX), *Thermus thermophilus* ADC (TthADC) (1VC3), TthADC, complexed with substrate analog fumarate (2EEO), *Helicobacter pylori* ADC (HpyADC) (1UHD) [15] and HpyADC, complexed with substrate analog isoasparagine (1UHE) [15]. The ADC protein folds into a double-ψ β-barrel structure. It forms a homotetramer [13] and the active site is shown to be at the interface of a dimer of processed ADC [15].

The unique feature of being absent in human, in addition to its significance in the cellular metabolism of Mtb, endows exclusive significance upon ADC as an important drug and vaccine target. Jacobs and coworkers [16] constructed a double deletion mutant (Δ*panCD*) with a view to globally impair the ability of Mtb to synthesize lipids. Mice infected with the Δ*panCD* mutant were able to survive 22 weeks longer than those infected with the bacille Calmette-Guerin-Pasteur (BCG-P) strain. Deletion of the genes significantly attenuates Mtb and protects infected animals against tuberculosis.

In an effort to discover inhibitors against ADC, β-hydroxyaspartate, L-cysteic acid, D-serine, oxaloacetate and succinic dehydrazine are reported as competitive inhibitors of ADC with K_i_ of 0.13, 0.08, 0.16, 0.81, 0.73 mM, respectively [17], [18], [19] and phenylhydrazine binds to the pyruvoyl group to inactivate the protein [17]. While D-serine, β-hydroxyaspartate, and L-cysteic acid interfere *in vivo* with the synthesis of pantothenic acid in bacteria, external supply of aspartic acid, β-alanine, or pantothenic acid can reverse their growth inhibitory action in *E. coli* [20], [21]. As these molecules do not have suitable pharmacochemical and pharmacokinetic properties (unpublished data), recent studies have emphasized the need to discover novel selective drug-like inhibitors against MtbADC [13], [22]. However, to date no selective drug-like inhibitor against MtbADC has been reported. To our knowledge, this is the first chemoinformatics-based drug design approach to propose novel and selective inhibitors of MtbADC.

## Materials and Methods

### Preparation of the processed MtbADC structure

Structural alignment of unprocessed and processed *E. coli* ADC structures [12] by the use of Multiprot [23] shows a root mean square deviation (RMSD) of 0.19 Å for 89 Cα atom pairs. This suggests that the unprocessed and processed ADC structures are highly similar. Thus, in preparation for virtual screening, unprocessed MtbADC (2C45) was modified to the processed form, in which Ser25 was substituted with a pyruvoyl group. The active site as well as conserved and functionally important residues were selected by structural alignment of the processed MtbADC with processed TthADC:fumarate and HpyADC:isoasparagine complex structures using Multiprot [23] and visualized using PYMOL [24]. As the active site is at a dimer interface, an appropriate dimer was prepared. The model was further refined by adding missing hydrogens, assigning proper bond orders, and was submitted to a series of restrained and partial minimization using the optimized potentials for liquid simulations all-atom (OPLS_AA) force field [25] in the Protein Preparation Wizard of Schrödinger [26].

### Structure-based virtual screening

To identify inhibitors against the above processed MtbADC, flexible ligand based high-throughput virtual screening (HTVS) mode of Glide 5.5 [27] was carried out using 333,761 molecules of commercially available ligands from the Maybridge (14,400 molecules; www.maybridge.com) and Zinc [28] [zinc.docking.org, including National Cancer Institute (hereafter NCI; 316,181 molecules) and the United States of America Foods and Drug Administration approved drugs (hereafter FDA; 3,180 molecules)] databases. Using the TthADC:fumarate crystal structure as a guide, fumarate was docked with processed MtbADC and the docking score was used as a reference to identify drug-like inhibitors. The Maybridge, NCI and FDA molecules, as well as fumarate, were prepared by accounting for missing hydrogens, possible ionized states, tautomers and low energy ring conformations using the Glide LigPrep application [26].

A grid file was generated using the Receptor Grid Generation protocol with centroid at the active site of the enzyme. A scaling factor of 1.0 was set to van der Waals (VDW) radii for the atoms of residues that presumably interact with ligands and the partial atomic charge was set to less than 0.25. Ligands were then allowed to dock with the high throughput screening (HTVS) mode and all the obtained molecules were subjected to the Glide extra precision (XP) mode of docking, which performs extensive sampling and provides reasonable binding poses [27]. At this stage, ligands were accepted only if: (i) they interacted with the residues that bind substrate analogs in the TthADC:fumarate and HpyADC:isoasparagine complex structures, and (ii) the binding affinity glide scores (G-scores) were better than the reference MtbADC:fumarate score. These ligands were further assessed for their drug-like properties based on Lipinski's rule of five [29] and also the absorption, distribution, metabolism, excretion and toxicity (ADMET) properties, calculated with QikProp version 3.2 (Schrödinger) [30]. In addition, the docking poses and structural properties of some of the known ADC inhibitors, phenylhydrazine (PubChem chemical database ID CID7516), L-cysteic acid (CID72886), Oxaloacetate (CID164550) and D-serine (CID71077), were compared with those of the selected drug-like molecules. As the active site of MtbADC is located at the interface of a dimer, the selected molecules were cross-verified by performing virtual screening against processed monomeric ADC.

### Non cross-reactivity with human pyruvoyl-dependent enzymes

The mechanism of action of the ADC protein is similar to other pyruvoyl dependent enzymes, such as histidine decarboxylase, S-adenosylmethionine (SAM) decarboxylase and phosphatidyl serine decarboxylase, which catalyze reactions utilizing the pyruvoyl residue as a prosthetic group [17], [31]. As of now, only the crystal structure of human SAM decarboxylase (PDB id: 3H0W) [32] is available in the PDB. In order to avoid any potential side-effect and cross reactivity of lead molecules with SAM decarboxylase, the inhibitors from the previous step that interacted with the pyruvoyl group or conserved substrate binding residues Glu247, Phe223, Phe7 or Glu67 (SAM decarboxylase numbering) were rejected. Furthermore, the inhibitors were checked for suitable ADMET properties and accepted.

## Results

### Structural overview of L-asparate α-decarboxylase

**Figure 1:**
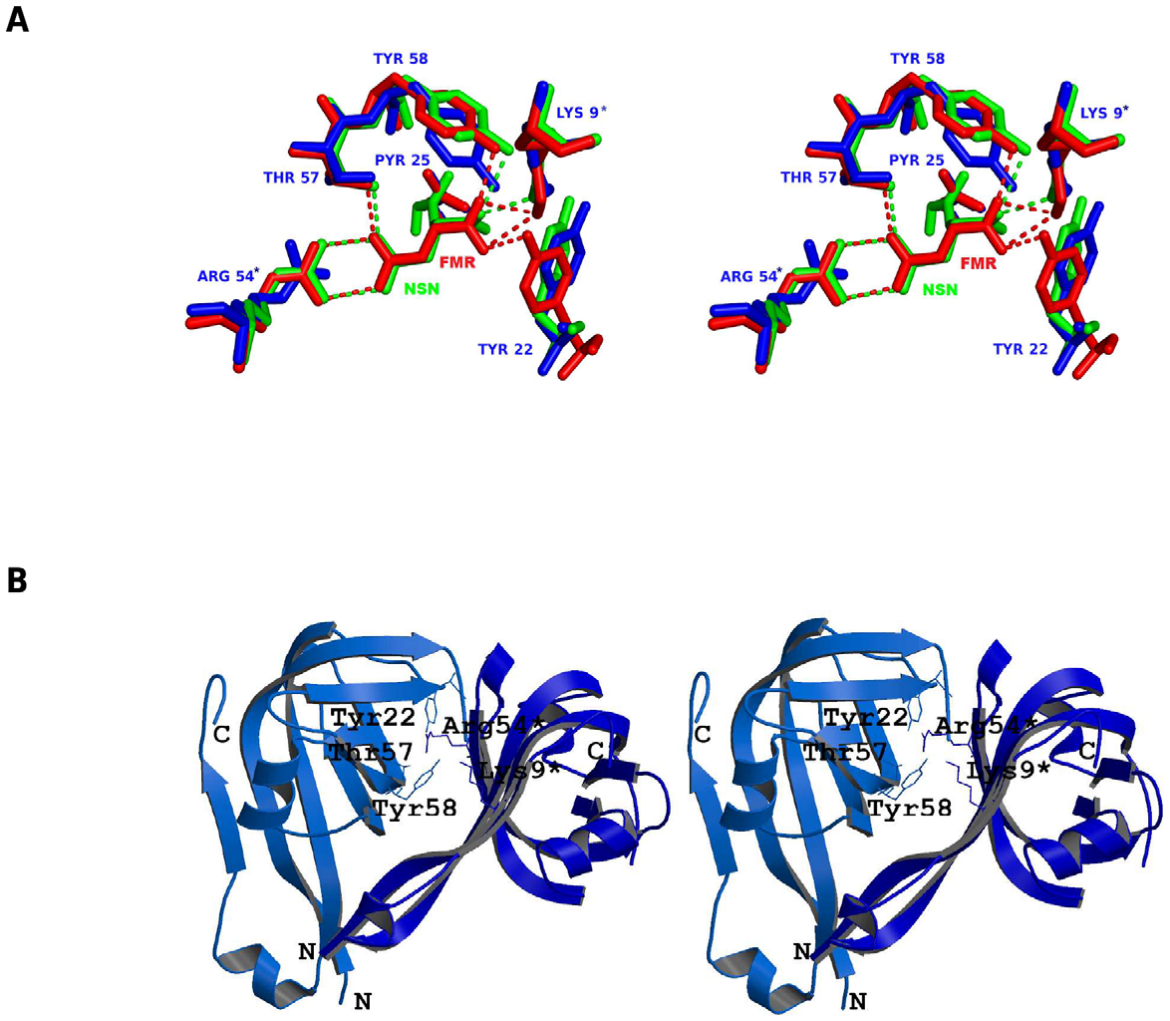
Conserved functional residues of ADCs that bind to substrate. (**a**) Stereo view of structural superimposition of processed MtbADC (blue), processed *Thermus thermophilus* ADC complexed with substrate analog fumarate (red and PDB id: 2EEO) and *Helicobacter pylori* ADC complexed with substrate analog isoasparagine (green, PDB id: 1UHE). The conserved and interacting residues are labeled according to MtbADC and the interactions are shown as dashed lines. (**b**) Stereo view of the active site in the dimer interface. The figure was prepared using Molscript [36] and Raster3D [37].

Gopalan and co-workers have solved the crystal structure of uncleaved MtbADC (2C45) at 2.99 Å resolution [13].The structural superimposition of processed MtbADC, TthADC:fumarate complex and HpyADC:isoasparagine complex shows that both substrate analogs bind to the active site (Fig. 1a), which is formed at the interface of a dimer in a similar orientation and are surrounded by conserved residues (Fig. 1b). The three proteins align with pair-wise RMSDs of less than 1 Å. Also, the binding of the substrate analogs to TthADC and HpyADC does not significantly change the structure of the enzyme which is evident from the RMSD values of 0.19 and 0.13 Å for 95 Cα atom pairs, respectively. Sequence and structural analyses reveal that the strictly conserved residues among ADCs are Lys9, His11, Tyr22, Gly24, Pyr25, Arg54, Thr57, Tyr58, Gly73, Ala74, Ala75, Ile86 and Asn112 (MtbADC numbering). The substrate analogs interact with Lys9*, Pyr25, Arg54*, Thr57, Tyr58 (* represents a residue from another subunit of the dimer), where Lys 9* keeps the α-carboxyl group of the substrate deprotonated by forming an ion pair [15], and Pyr25 is a cofactor responsible for the activity of the enzyme [12]. Furthermore, Arg54* contributes to substrate specificity [15], Thr57 interacts with substrate and plays an important role in catalysis [15] and Tyr58 acts as a proton donor in the decarboxylation reaction [33].

### Selection of inhibitors

Binding of a ligand to at least one conserved functional residue is likely to interfere with the binding or catalysis of substrate and would result in inhibition of the function of the protein. Out of 333,761 molecules from the three public ligand databases, 190 hits from the Maybridge, 473 hits from NCI and 140 hits from FDA databases were initially obtained with the high throughput virtual screening (HTVS) mode. The ligands were allowed to bind with processed MtbADC in the more precise Glide extra-precision mode (Glide XP) and subsequently 28 ligands (3 Maybridge, 7 NCI and 18 FDA) with binding energy (−4.9 kcal/mol or higher) better than that for fumarate (−4.2 kcal/mol) were selected. These ligands interact with at least one of the experimentally determined conserved functional residues (Table S1).

Of 28, seven ligands (5 FDA and 2 NCI) interact with the MtbADC pyruvoyl group, suggesting that they could cross-react with other pyruvoyl dependent enzymes. An assessment of cross-reactivity of the putative inhibitors with the human SAM decarboxylase structure, a representative of pyruvoyl dependent enzymes, identified that only one (ZINC03871163) of the seven molecules does not interact with any of the conserved substrate binding residues Glu247, Phe223, Phe7 or Glu67 (SAM decarboxylase numbering) or the pyruvoyl group of SAM decarboxylase.

**Figure 2:**
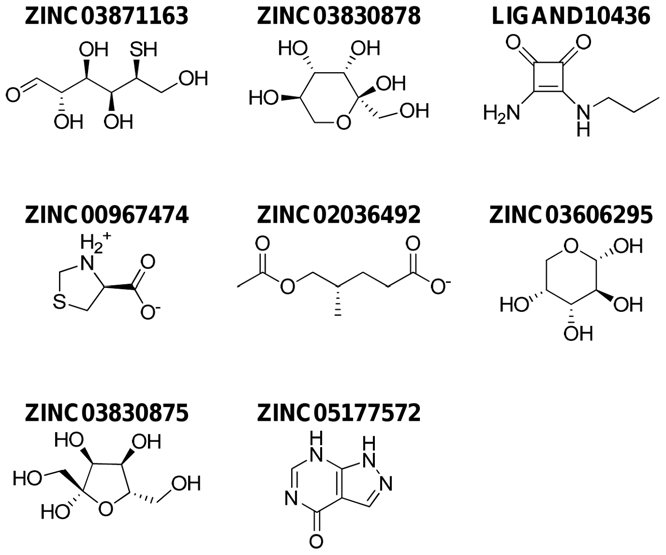
Chemical structures of the eight lead molecules. ZINC03871163: (2S,3R,4S,5S)-2,3,4,6-tetrahydroxy-5-mercaptohexanal, ZINC03830878: (2S,3S,4S,5R)-2(hydroxymethyl) tetrahydro-2H-pyran-2,3,4,5-tetraol, LIGAND10436: 3-amino-4-(propylamino)cyclobutane-1,2-dione, ZINC00967474: (S)-thiazolidin-3-ium-4-carboxylate, ZINC02036492: (S)-5-acetoxy-4-methylpentanoate,ZINC03606295:(2S,3S,4R,5R)tetrahydro2Hpyran2,3,4,5tetraol,ZINC03830875:(2S,3S,4R,5S)2,5bis(hydroxymethyl)tetrahydrofuran-2,3,4-triol,ZINC05177572:1H-pyrazolo[3,4-d]pyrimidin-4(7H)-one.

Eight lead molecules significantly satisfy the pharmacokinetic factors that are defined for human use and qualify as potential drug-like molecules. They are: (2S,3R,4S,5S)-2,3,4,6-tetrahydroxy-5-mercaptohexanal (ZINC03871163), (2S,3S,4S,5R)-2 (hydroxymethyl) tetrahydro-2H-pyran-2,3,4,5-tetraol (ZINC03830878), 3-amino-4-(propylamino) cyclobutane-1,2-dione (LIGAND10436), (S)-thiazolidin-3-ium-4-carboxylate (ZINC00967474), (S)-5-acetoxy-4-methylpentanoate (ZINC02036492), (2S,3S,4R,5R)-tetrahydro-2H-pyran-2,3,4,5-tetraol (ZINC03606295), (2S,3S,4R,5S)-2,5-bis(hydroxymethyl)tetrahydrofuran-2,3,4-triol (ZINC03830875) and 1H-pyrazolo [3,4-d]pyrimidin-4(7H)-one (ZINC05177572). The interacting residues for these lead molecules are shown in Table 1 and their structures are shown in Fig. 2.

**Table 1:** Glide extra-precision (XP) results for the eight lead molecules and fumarate by Schrodinger 9.0.

Ligand IDs^a^	Gscore^b^	Interacting amino acids^c^					H^d^	Database^e^
3871163	−6.05	Asn72 (1.6)	Gly73(1.83)	Pyr25 (2.1)	Tyr22 (1.95)	Arg12(1.95)	5	NCI
3830878	−6.03	Asn72 (1.93)	Arg54(2.27)	Asn72(1.78)			2	FDA
10436	−6.00	Tyr58 (1.88)	Gly73 (1.69)	Asn72 (1.76)			3	Maybridge
967474	−5.42	Tyr58 (1.8)	Asn72(1.6)				2	FDA
2036492	−5.29	Thr57(1.8)	Tyr58 (2.1)	Lys9(2.1)			3	NCI
3606295	−5.11	Arg54 (2.12)	Thr57 (1.74)	Tyr58 (2.1)	Asn72 (1.67)	Gly73 (1.69)	5	FDA
3830875	−4.97	Arg54(2.3)	Thr57 (2.29)	Asn72 (2.25)	Asn72 (1.74)		3	FDA
5177572	−4.96	Asn72 (1.7)	Tyr58 (2.1)	Arg54 (2.0)			3	FDA
Fumarate	−4.20	Thr57(1.7)	Lys9(2.34)	Tyr58 (1.60)	Arg54(2.16)		4	

a The ids of the lead molecules.

b Glide extra precision scores (kcal/mol).

c The residues interacting with the lead molecules. The value in bracket represents the hydrogen bond distance between the atoms of respective residues, in angstrom (A°).

d H represents the number of hydrogen bond.

e The ligand belong to the corresponding chemical database.

**Figure 3:**
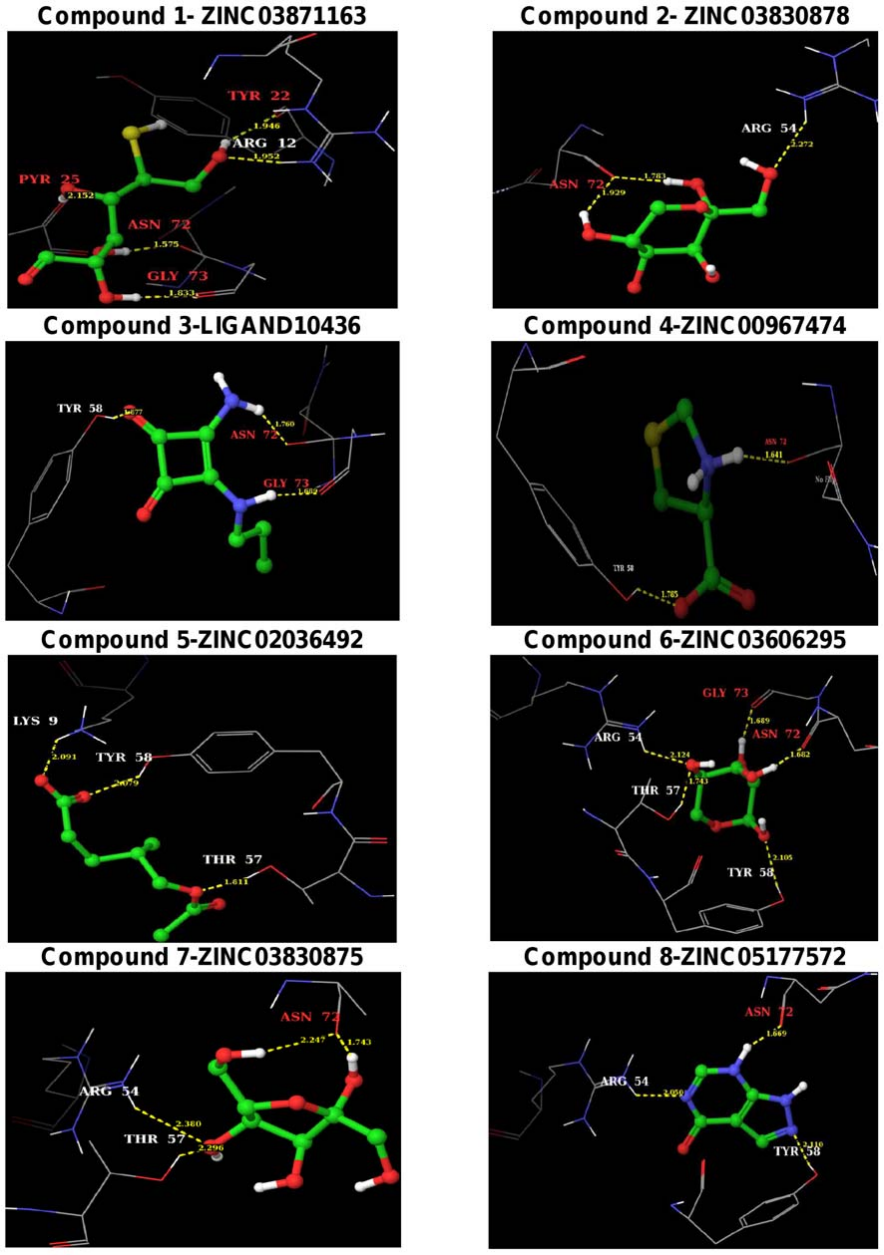
Binding poses of the identified eight lead molecules with MtbADC. The binding modes of the proposed lead molecules are shown as ball and stick. Atoms colors are: H: white, C: green, N: blue, O: red and S: yellow. The interacting MtbADC residues are drawn as thin wireframe in the same color scheme and are labeled. Hydrogen bond interactions are shown as dotted yellow lines, along with the distance between donor and acceptor atoms. The binding pose of protein:lead molecule interactions were generated with the Maestro program in the Schrodinger software suite.

The pharmacokinetic properties of the 28 ligands were assessed by the use of Qikprop (Table S2). The above eight lead molecules (Table 2) fulfill drug-like properties based on Lipinski's rule of five. The molecular weight of the lead molecules are less than 500 kDa, number of hydrogen bond donors is less than 5 and hydrogen bond acceptors are less than 10. The predicted octanol/water partition coefficient (QPlogPo/w) and aqueous solubility (QPlogS) are in the acceptable range i.e. −2.0 to 6.5 and −6.5 to 0.5, respectively. The predicted IC_50_ value for the blockage of HERG K^+^ channels (QPlogHERG) is in the acceptable range of above −5.0. The cell permeability (QPPCaco), a factor that is responsible for drug metabolism and its access to the biological membrane, is within the acceptable range 25 to 500. As the cell wall of *Mycobacterium tuberculosis* is thicker than that of other bacteria, the selected inhibitors must have a reasonable cell permeability value to cross the membrane. The predicted value of binding to human serum albumin (QPkhsa) is within the acceptable limit −1.5 to 1.5. Absorption is one of the important pharmacokinetic factors, especially when the most convenient way of drug administration is oral consumption. Percentage of human oral absorption is also within the acceptable range (<25% is poor and >80% is high). The selected inhibitors form 2 to 5 hydrogen bond contacts with the conserved residues of MtbADC, with glide XP scores of −5 to −6 kcal/mol, which are superior to the −4.0 kcal/mol score of fumarate. The hydrogen bond distances range from 1.6 to 2.3 Å, suggesting strong ligand-MtbADC interaction. The binding pose of the selected eight lead molecules are illustrated in Fig. 3.

**Table 2:** Assessment of drug-like properties of the lead molecules and fumarate as verified by Qikprop (Schrodinger 9.0).

Ligand IDs^a^	MW^b^	HD^c^	HA^d^	QPlogPo/w^e^	QPlogS^f^	QPlogHERG^g^	QPPCaco^h^	Percent human oral absorption^i^	QPlogKhsa^j^
3871163	196.2	3.8	8.3	−1.3	−0.4	−2.9	89.1	54.2	−1.1
3830878	180.2	5.0	8.3	−1.7	−0.8	−2.5	106.9	53.3	−0.9
10436	154.2	3.0	6.0	−0.9	−1.1	−3.3	125.6	59.5	−0.8
967474	133.2	2.0	4.0	−2.0	−0.4	−1.4	41.9	44.1	−0.9
2036492	174.2	1.0	4.0	1.2	−1.7	−1.5	74.8	67.7	−0.7
3606295	150.1	4.0	8.5	−1.7	−0.9	−2.1	192.4	57.7	−0.8
3830875	180.2	5.0	8.3	−1.7	−0.7	−2.7	106.4	53.3	−0.9
5177572	136.1	2.0	4.5	−0.6	−2.3	−2.9	166.7	63.5	−0.7
Fumarate	114.1	2.0	4.0	−0.3	−0.2	0.8	4.1	36.5	−1.2

a Ligand IDs are of the Maybridge, NCI, FDA ligand databases.

b Molecular weight (<500 Da).

c Hydrogen bond donors (<5).

d Hydrogen bond acceptors (<10).

e Predicted octanol/water partition co-efficient log p (recommended range: −2.0 to 6.5).

f Predicted aqueous solubility; S in mol/L (acceptable range: −6.5 to 0.5).

g Predicted IC_50_ value for blockage of HERG K+ channels (acceptable range: above −5.0).

h Predicted Caco-2 cell permeability in nm/s (acceptable range: 25 is poor and .500 is great).

i Percentage of human oral absorption (<25% is poor and >80% is high).

j Prediction of binding to human serum albumin (acceptable range: −1.5 to 1.5).

### *In silico* validation

The reliability of the docking protocol was evaluated by comparing the crystallographic pose of the TthADC:fumarate complex structure with the processed MtbADC:fumarate docking model, obtained from the Glide XP mode. The docking procedure precisely predicted the experimental result. This indicates that the Glide docking protocol represents nearly the same binding conformation that is present in native crystal structure. In both cases, fumarate binds to the same binding site and interacts with residues, Lys9*, Arg54*, Thr57 and Tyr58 (Fig. S2).

The ADC active site is formed by the interface of a dimer, with relatively small volume. This cleft can support only molecules of relatively small size [34]. The reliability of the identified drug-like lead molecules is justified by the fact that they are small in size, when compared to some known ADC inhibitors (Figs. S3 and S4). To further support our results, fumarate was docked with the MtbADC monomer and dimer. It binds to the ADC monomer with −1.5 kcal/mol glide score, whereas docking to the ADC dimer gives a Gscore of −4.2 kcal/mol in the Glide XP mode. This clearly underlines the fact that the active site, formed by the dimer interface, favors the binding of only small ligands. Furthermore, the conserved functional residue Arg54, which is responsible for substrate specificity [15], is brought to the proximity of the substrate binding site only in the dimeric conformation. Our results were cross checked with the binding modes of the known ADC inhibitors, which were modeled using docking studies. These inhibitors bind to the same site as fumarate and isoasparagine, and interact with at least one of the conserved experimentally determined functional residues (Table S3 and Fig. S5). In vitro experimental verification of the 28 inhibitors of Table S1 and the inhibitors reported in the literature are underway (unpublished data). Furthermore, shortlisted inhibitors will be verified in *Mycobacterium tuberculosis* culture using established techniques and a latest protocol [35].

## Discussion

The present work identifies eight drug-like lead molecules, from three available public ligand databases, that interact with the known functionally conserved residues of aspartate α- decarboxylase. These residues have experimentally been shown to interact with substrate analogs fumarate and isoasparagine. In addition, the lack of cross-reactivity of these inhibitors with pyruvoyl dependent S-adenosylmethionine (SAM) decarboxylase suggests that these lead molecules can be potential selective drug-like inhibitors of MtbADC. Our findings are consistent with known experimental results and support the idea that the active site is at the dimer interface for substrate or inhibitor specificity. The backbone scaffolds of the identified eight drug-like molecules can provide suitable platforms for the development of potential therapeutics, after adequate experimental follow up, in the treatment of tuberculosis.

The exact role of the conserved residue Tyr22 in catalysis is not clear as its flexible side chain is not oriented towards the substrate analog in the HpyADC:isoasparagine [15] complex. However, it binds to fumarate in the TthADC:fumarate complex structure. To understand its role, an alternate rotamer was chosen in our chemoinformatics approach by placing it towards the active site, mimicking the TthADC:fumarate complex structure. To our surprise, the glide score was better with −5.27 kcal/mol (instead of −4.2 kcal/mol), representing a favorable binding mode. The analysis of this binding pose suggests the interaction of ligands with Tyr22 may follow the TthADC:fumarate complex structure. However, this is in contrast to the HpyADC:isoasparagine complex structure, where Tyr22 does not interact with the substrate analog. Hence, the role of Tyr22 needs to be further verified with additional mutational, structural and biochemical experiments.

The central dogma for the identification of new anti-TB drugs is that the inhibitors should be active against both latent (or dormant) and non-dormant bacilli. Mtb, as a pathogen, has evolved to exploit dormancy or latency to its advantage. Since most of the current drugs target the rapidly growing organism, the current drug regimens in the treatment of this infection tend to be of extraordinarily long durations, which lead to tremendous problems, like patient noncompliance and development of MDR strains. This is where the uniqueness of targeting ADC as a drug target may be of considerable importance. An inhibitor or drug that is developed against ADC could be used in conjunction with conventional antibiotics. This combined attack can shorten the time frame of the current therapy and help for an effective clearance of the bacterium from patients.

## Supporting Information

Figure S1 **Pantothenate and CoA biosynthesis pathway.** L-Aspartate α-decarboxylase (ADC) catalyzes the decarboxylation of L-aspartate to β-alanine.
(TIFF)

Figure S2 **Fumarate binding in ADC.** (**a**) The superimposed view of the TthADC:fumarate crystal structure (red) and MtbADC:fumarate docking model (blue) is shown. The processed model for MtbADC was generated from the crystal structure of unprocessed protein and docking of fumarate was achieved using the Glide Extra Precision mode. The interacting conserved residues are labelled for MtbADC and the interactions between the protein and fumarate are shown as dashed lines in the corresponding colors. (**b**) Surface diagram around the substrate binding cavity in the same orientation of panel A. The interacting protein residues are also shown in faint trace.
(TIFF)

Figure S3 **The structures of known and reported inhibitors against ADC.**
(TIFF)

Figure S4 **Ligands docked to monomeric MtbADC.** The structures of the top three hits, obtained by docking the Maybridge, NCI and FDA databases with the processed monomeric MtbADC structure. These molecules are big and cannot be genuine inhibitors as the actual active site is formed in the cleft of a dimer with relatively smaller volume and only molecules of small size can bind in the pocket.
(TIFF)

Figure S5 **Binding poses of known inhibitors/ligands.** The known inhibitors or ligands are shown as thick ball and stick. Atoms are colored as: H: white, C: green, N: blue, O: red and S: yellow. The interacting MtbADC residues are drawn as thin wireframe with the same color scheme and are labeled. Hydrogen bond interactions are shown as dotted yellow lines, along with the distance between donor and acceptor atoms.
(TIFF)

Table S1 **The 28 ligand hits from the Maybridge, NCI and FDA databases which interact with at least one of the conserved functional residues of MtbADC residues involved in substrate binding and their glide score (kcal/mol).** The ligands are ranked according to their glide scores in their respective databases. The ligands that interact with Pyr25 are in bold. The entries of Table 1 are underlined.
(DOCX)

Table S2 **The ADMET properties of the 28 ligands.** The ligands that interact with Pyr25 are in bold. The entries of Table 2 are underlined. The definitions of the properties are as in Table 2.
(DOCX)

Table S3 **Interactions of selected known inhibitors/ligands with MtbADC as verified by Glide XP.**
(DOCX)
