# Supplementary figures and images for: Chemoinformatic Identification of Novel Inhibitors against Mycobacterium tuberculosis L-aspartate α-decarboxylase
Source: PLoS One. 2012 Mar 28;7(3):e33521. doi: 10.1371/journal.pone.0033521 (PMC3314653; doi:10.1371/journal.pone.0033521)

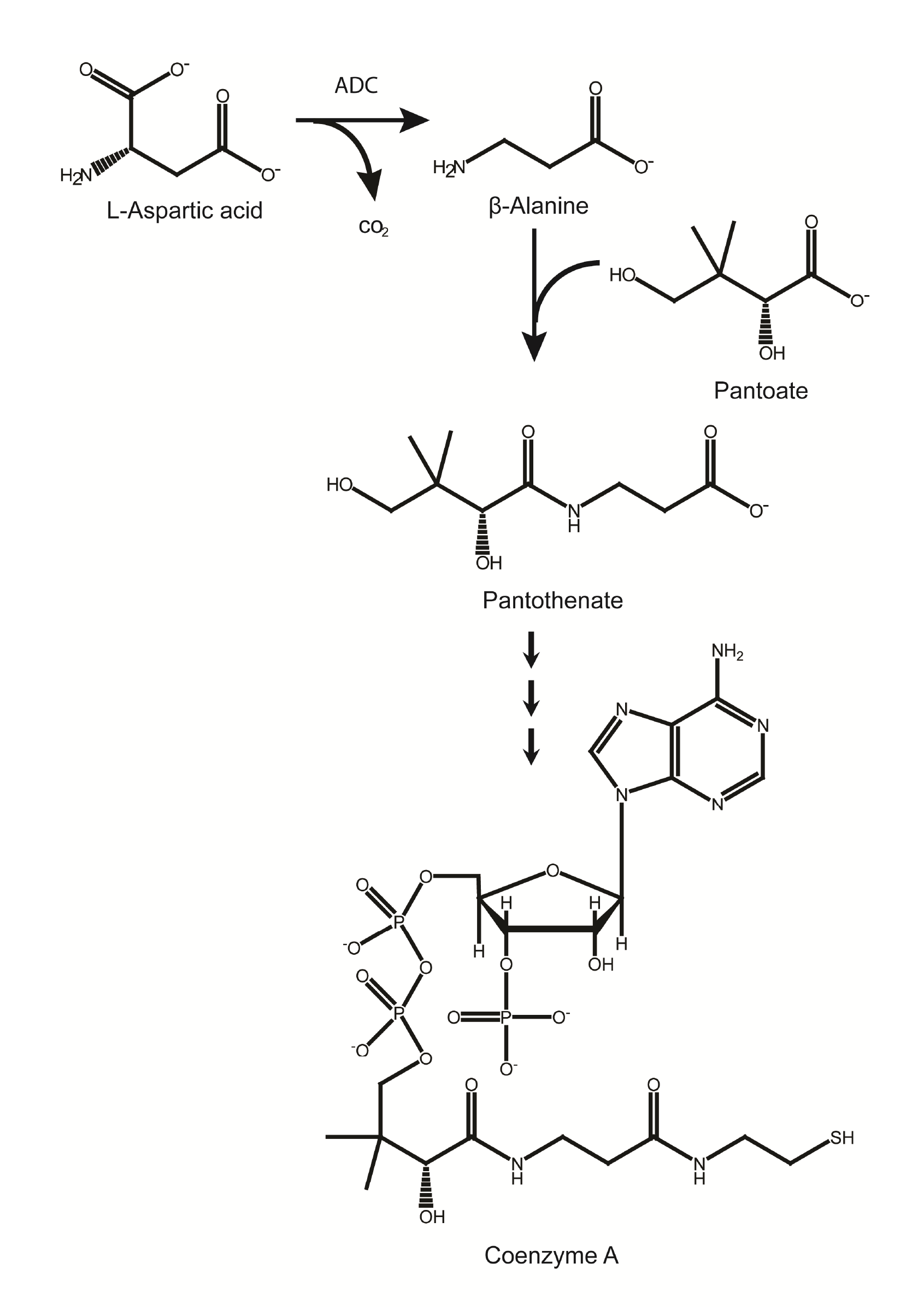

Supplement: Figure S1 — Pantothenate and CoA biosynthesis pathway. L-Aspartate α-decarboxylase (ADC) catalyzes the decarboxylation of L-aspartate to β-alanine. (TIFF) [file pone.0033521.s001.tiff]

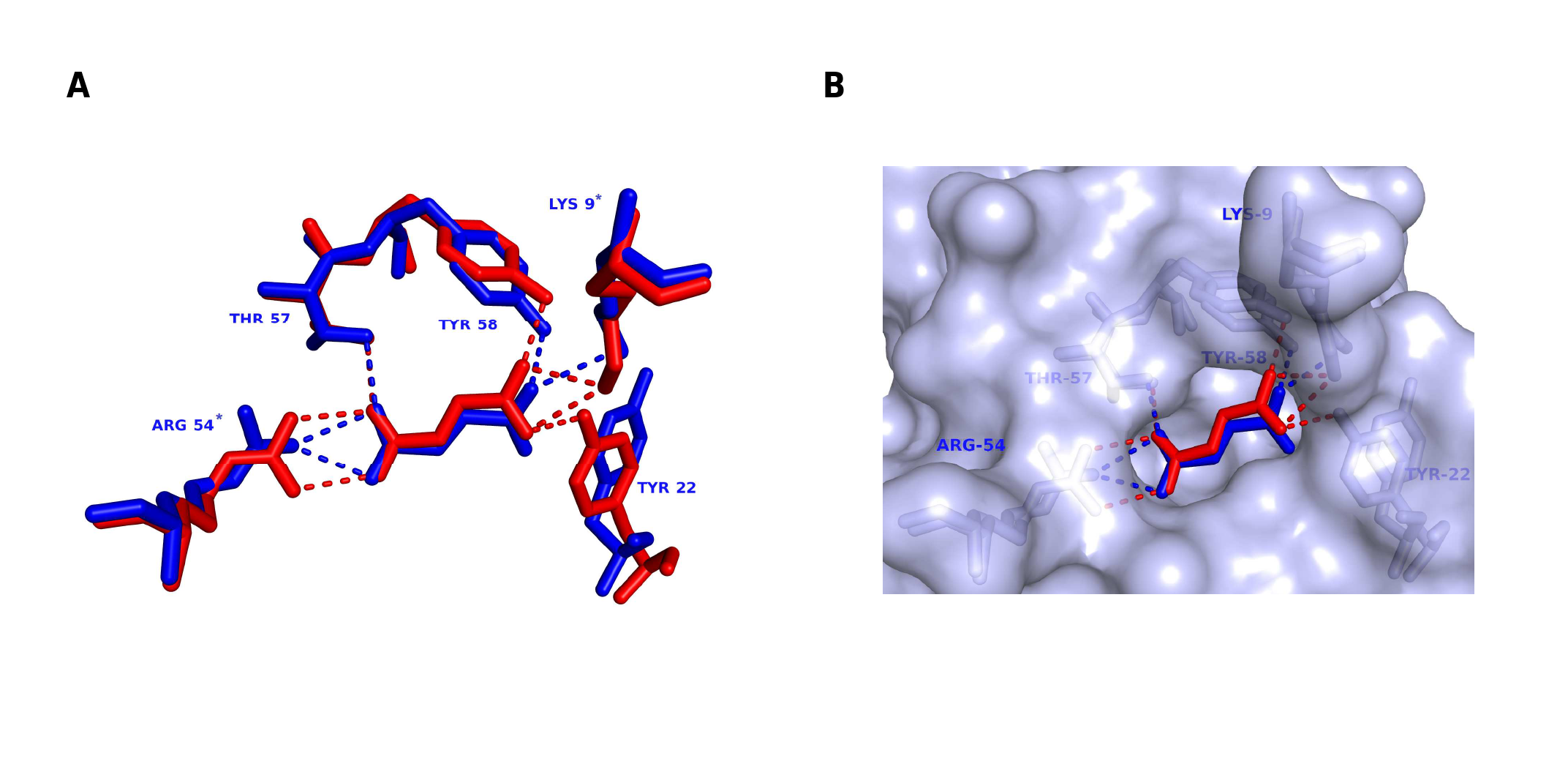

Supplement: Figure S2 — Fumarate binding in ADC. (a) The superimposed view of the TthADC:fumarate crystal structure (red) and MtbADC:fumarate docking model (blue) is shown. The processed model for MtbADC was generated from the crystal structure of unprocessed protein and docking of fumarate was achieved using the Glide Extra Precision mode. The interacting conserved residues are labelled for MtbADC and the interactions between the protein and fumarate are shown as dashed lines in the corresponding colors. (b) Surface diagram around the substrate binding cavity in the same orientation of panel A. The interacting protein residues are also shown in faint trace. (TIFF) [file pone.0033521.s002.tiff]

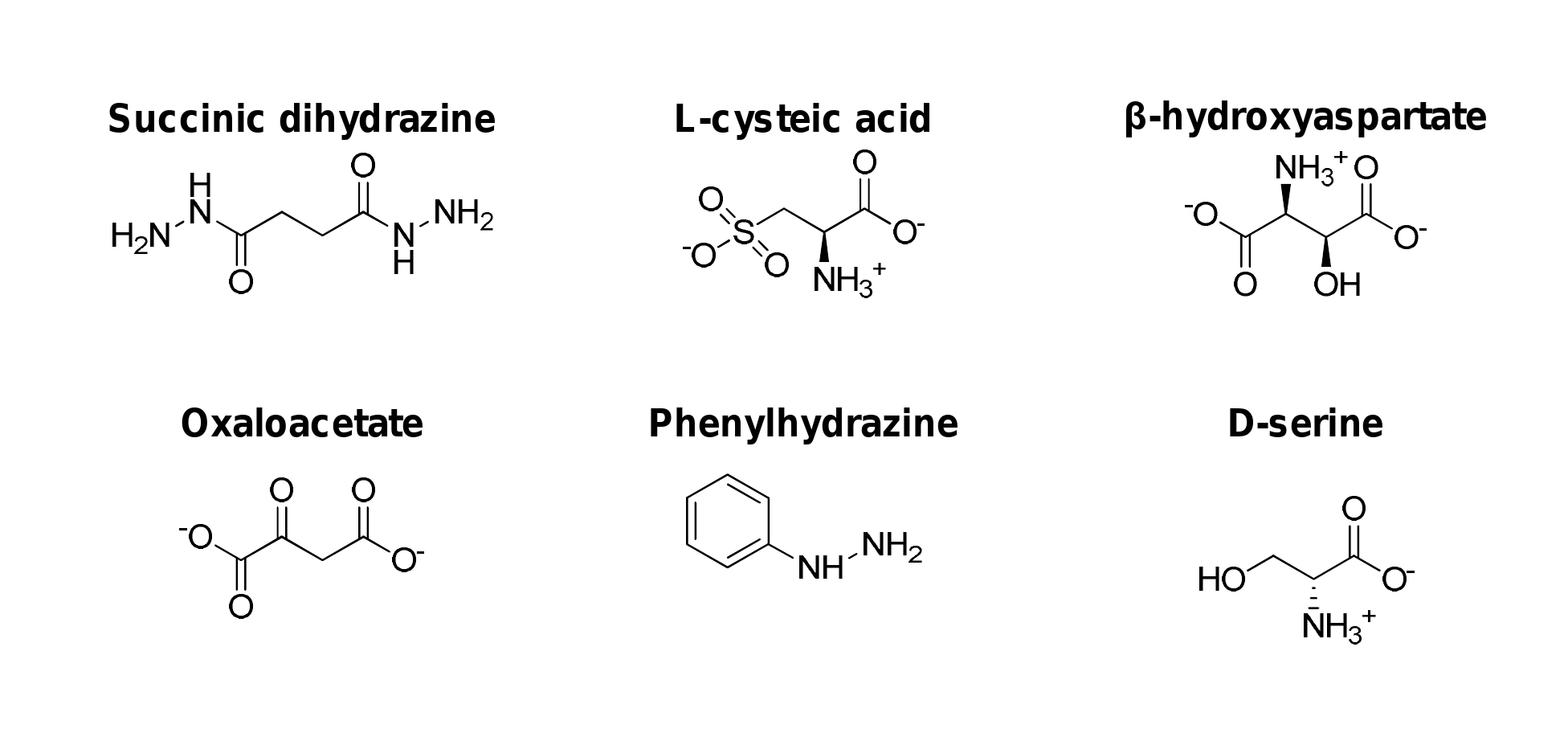

Supplement: Figure S3 — The structures of known and reported inhibitors against ADC. (TIFF) [file pone.0033521.s003.tiff]

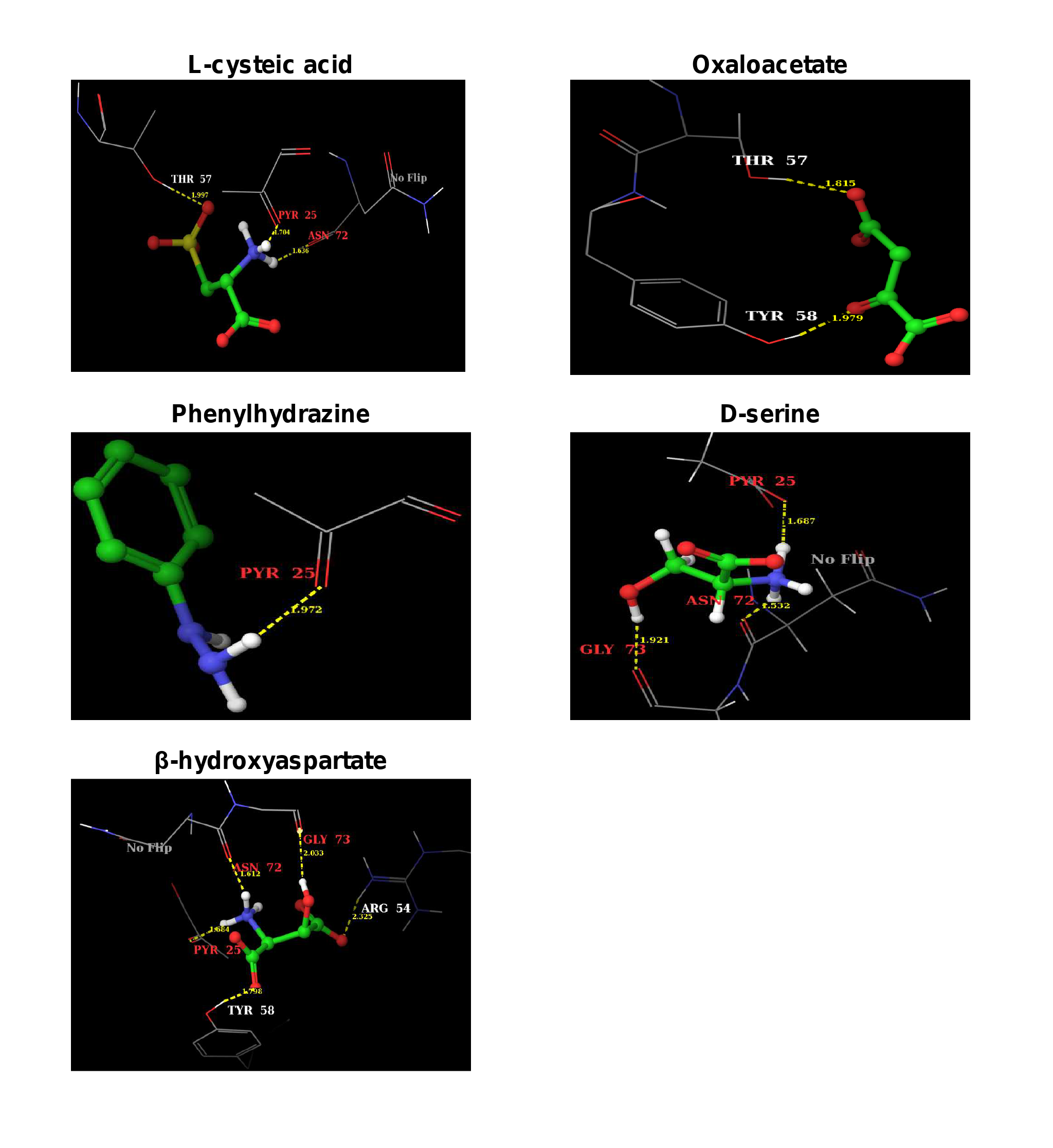

Supplement: Figure S4 — Ligands docked to monomeric MtbADC. The structures of the top three hits, obtained by docking the Maybridge, NCI and FDA databases with the processed monomeric MtbADC structure. These molecules are big and cannot be genuine inhibitors as the actual active site is formed in the cleft of a dimer with relatively smaller volume and only molecules of small size can bind in the pocket. (TIFF) [file pone.0033521.s004.tiff]

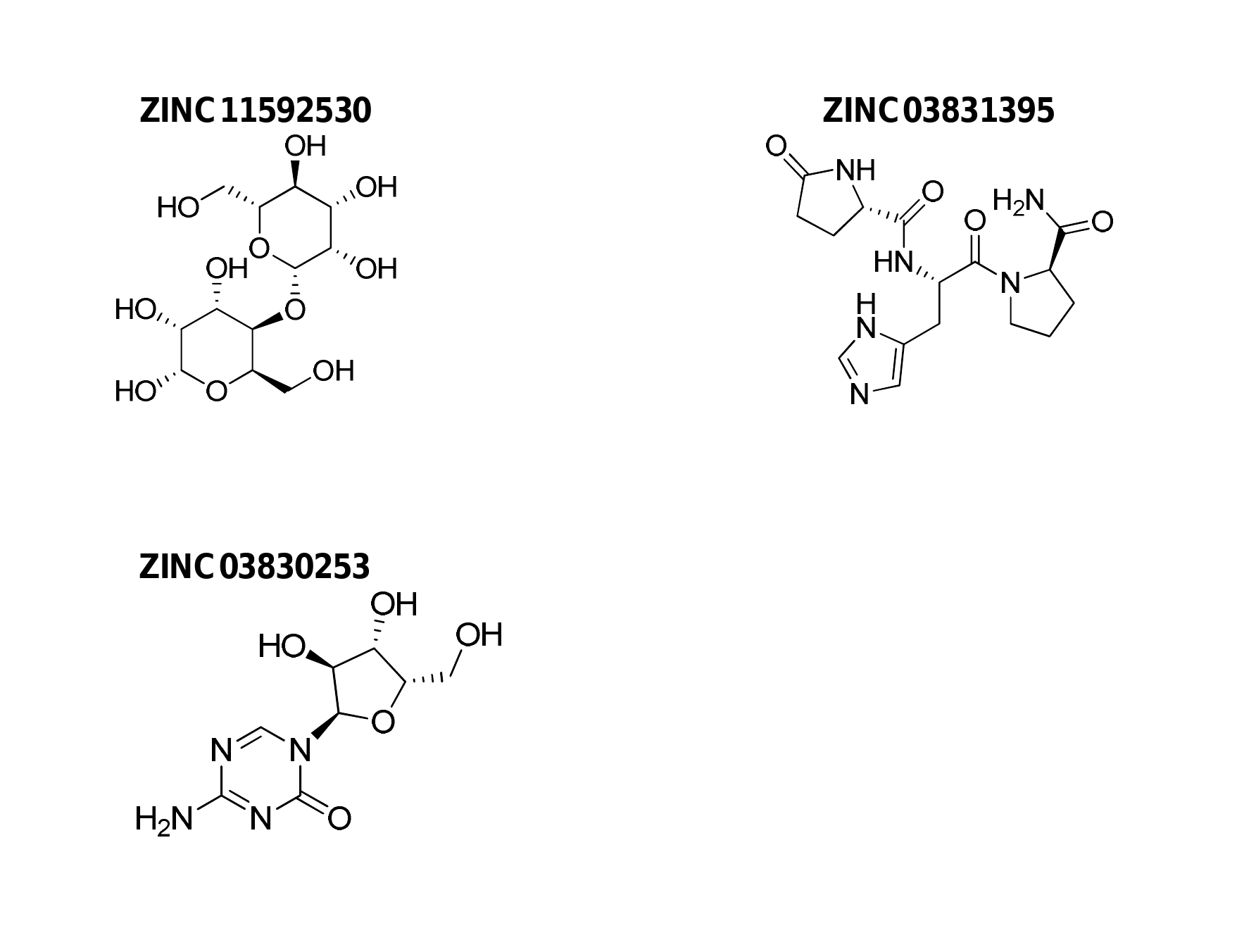

Supplement: Figure S5 — Binding poses of known inhibitors/ligands. The known inhibitors or ligands are shown as thick ball and stick. Atoms are colored as: H: white, C: green, N: blue, O: red and S: yellow. The interacting MtbADC residues are drawn as thin wireframe with the same color scheme and are labeled. Hydrogen bond interactions are shown as dotted yellow lines, along with the distance between donor and acceptor atoms. (TIFF) [file pone.0033521.s005.tiff]
