# Supplementary material for: Chemoinformatic Identification of Novel Inhibitors against Mycobacterium tuberculosis L-aspartate α-decarboxylase
Source: PLoS One. 2012 Mar 28;7(3):e33521. doi: 10.1371/journal.pone.0033521 (PMC3314653; doi:10.1371/journal.pone.0033521)
Supplement: Table S1 — The 28 ligand hits from the Maybridge, NCI and FDA databases which interact with at least one of the conserved functional residues of MtbADC residues involved in substrate binding and their glide score (kcal/mol). The ligands are ranked according to their glide scores in their respective databases. The ligands that interact with Pyr25 are in bold. The entries of Table 1 are underlined. (DOCX) [file pone.0033521.s006.docx]

### Chemoinformatic identification of novel inhibitors against *Mycobacterium tuberculosis* L-aspartate α-decarboxylase

Reetu Sharma, Roopa Kothapalli, Antonius M.J. Van Dongen and Kunchithapadam Swaminathan

**Supplementary tables**

**Table S1.** The 28 ligand hits from the Maybridge, NCI and FDA databases which interact with at least one of the conserved functional residues of MtbADC residues involved in substrate binding and their glide score (kcal/mol). The ligands are ranked according to their glide scores in their respective databases. The ligands that interact with Pyr25 are in bold. The entries of Table 1 are underlined.

| **Molecule ID** | **Glide score** | **Interacting residues** | | | | |
| --- | --- | --- | --- | --- | --- | --- |
| **FDA database** |  |  |  |  |  |  |
| ZINC00895296 | -6.783547 | Thr57 | Gly73 | Asn72 | Arg54 |  |
| **ZINC03831017** | -6.524427 | Pyr25 | Asn72 | Thr57 |  |  |
| ZINC02556854 | -6.378547 | Asn72 | Gly73 | Thr57 |  |  |
| **ZINC02041302** | -6.352301 | Arg54 | Gly73 | Asn72 | Pyr25 | Tyr58 |
| **ZINC02507451** | -6.256358 | Asn72 | Gly73 | Arg54 | Pyr25 | Tyr58 |
| ZINC00895297 | -6.062757 | Asn72 | Thr57 | Tyr58 | Lys9 |  |
| ZINC01532640 | -6.057464 | Thr57 | Asn72 |  |  |  |
| ZINC03830878 | -6.028674 | Arg54 | Asn72 |  |  |  |
| ZINC12358606 | -5.884999 | Asn72 | Thr57 | Tyr58 | Lys9 |  |
| ZINC03831018 | -5.879572 | Arg54 | Asn72 | Thr57 | Tyr58 |  |
| **ZINC12362045** | -5.620917 | Gly73 | Asn72 | Pyr25 |  |  |
| ZINC00967474 | -5.424642 | Tyr58 | Asn72 |  |  |  |
| **ZINC03830688** | -5.11056 | Gly73 | Thr57 | Pyr25 |  |  |
| ZINC03606295 | -5.106771 | Arg54 | Thr57 | Asn72 | Tyr58 | Gly73 |
| ZINC01532526 | -5.001161 | Arg54 | Thr57 | Asn72 | Gly73 |  |
| ZINC03830875 | -4.965374 | Asn72 | Arg54 | Thr57 |  |  |
| ZINC05177572 | -4.9588 | Asn72 | Arg54 | Tyr58 |  |  |
| ZINC01529732 | -4.940066 | Arg54 | Thr57 | Tyr58 | Lys9 |  |
|  |  |  |  |  |  |  |
| **Maybridge database** |  |  |  |  |  |  |
| LIGAND10436 | -6.001754 | Tyr58 | Gly73 | Asn72 |  |  |
| LIGAND7497 | -5.43 | Asn72 | Thr57 | Arg54 |  |  |
| LIGAND6555 | -5.415633 | Asn72 | Arg54 | Tyr58 |  |  |
|  |  |  |  |  |  |  |
| **NCI Database** |  |  |  |  |  |  |
| ZINC18141652 | -6.667792 | Asn72 | Tyr58 |  |  |  |
| **ZINC03871163** | -6.049714 | Gly73 | Asn72 | Tyr22 | Arg12 | Pyr25 |
| ZINC00901606 | -5.77977 | Tyr58 | Gly73 | Thr57 | Arg54 |  |
| **ZINC01583698** | -5.709624 | Arg54 | Thr57 | Pyr25 | Tyr22 | Asn72 |
| ZINC08733367 | -5.669926 | Asn72 | Gly73 | Tyr58 |  |  |
| ZINC02597098 | -5.51695 | Arg54 | Thr57 | Tyr58 |  |  |
| ZINC02036492 | -5.286988 | Thr57 | Tyr58 | Lys9 |  |  |
