# Supplementary material for: Chemoinformatic Identification of Novel Inhibitors against Mycobacterium tuberculosis L-aspartate α-decarboxylase
Source: PLoS One. 2012 Mar 28;7(3):e33521. doi: 10.1371/journal.pone.0033521 (PMC3314653; doi:10.1371/journal.pone.0033521)
Supplement: Table S2 — The ADMET properties of the 28 ligands. The ligands that interact with Pyr25 are in bold. The entries of Table 2 are underlined. The definitions of the properties are as in Table 2. (DOCX) [file pone.0033521.s007.docx]

### Chemoinformatic identification of novel inhibitors against *Mycobacterium tuberculosis* L-aspartate α-decarboxylase

Reetu Sharma, Roopa Kothapalli, Antonius M.J. Van Dongen and Kunchithapadam Swaminathan

**Supplementary tables**

**Table S2.** The ADMET properties of the 28 ligands. The ligands that interact with Pyr25 are in bold. The entries of Table 2 are underlined. The definitions of the properties are as in Table 2.

| **Molecule ID** | **MW** | **HD** | **HB** | **QPlogPo/w** | **QPlogS** | **QPlogHERG** | **QPPCaco** | **Percent human oral absorption** |
| --- | --- | --- | --- | --- | --- | --- | --- | --- |
| **FDA database** |  |  |  |  |  |  |  |  |
| ZINC00895296 | 150.088 | 2 | 5.4 | -0.399 | -0.479 | 1.033 | 1.573 | 28.133 |
| **ZINC03831017** | 182.173 | 6 | 10.2 | -3.06 | -0.091 | -2.987 | 82.485 | 30.372 |
| ZINC02556854 | 182.173 | 6 | 10.2 | -3.08 | -0.081 | -2.954 | 62.848 | 28.137 |
| **ZINC02041302** | 182.173 | 6 | 10.2 | -3.099 | -0.07 | -3 | 49.602 | 26.19 |
| **ZINC02507451** | 307.966 | 4 | 6.8 | 0.252 | -1.433 | -3.38 | 513.35 | 76.934 |
| ZINC00895297 | 150.088 | 2 | 5.4 | -0.415 | -0.47 | 1.069 | 1.472 | 27.518 |
| ZINC01532640 | 182.173 | 6 | 10.2 | -3.093 | -0.074 | -2.989 | 55.067 | 27.033 |
| ZINC03830878 | 180.157 | 5 | 8.3 | -1.697 | -0.812 | -2.529 | 106.912 | 53.326 |
| ZINC12358606 | 150.088 | 2 | 5.4 | -0.415 | -0.47 | 1.069 | 1.472 | 27.518 |
| ZINC03831018 | 182.173 | 6 | 10.2 | -3.084 | -0.08 | -3.046 | 64.442 | 28.312 |
| **ZINC12362045** | 182.173 | 6 | 10.2 | -3.074 | -0.084 | -2.964 | 68.789 | 28.879 |
| ZINC00967474 | 133.165 | 2 | 4 | -2.033 | -0.416 | -1.401 | 41.887 | 44.073 |
| **ZINC03830688** | 307.966 | 4 | 6.8 | 0.253 | -1.378 | -3.278 | 538.306 | 77.308 |
| ZINC03606295 | 150.131 | 4 | 8.5 | -1.722 | -0.861 | -2.105 | 192.429 | 57.744 |
| ZINC01532526 | 146.146 | 5 | 5.5 | -4.215 | 1.237 | -0.419 | 1.03 | 2.491 |
| ZINC03830875 | 180.157 | 5 | 8.3 | -1.689 | -0.677 | -2.678 | 106.382 | 53.333 |
| ZINC05177572 | 136.113 | 2 | 4.5 | -0.551 | -2.262 | -2.942 | 166.698 | 63.49 |
| ZINC01529732 | 179.19 | 4 | 5.5 | -2.512 | -0.261 | -0.059 | 0.66 | 9.007 |
|  |  |  |  |  |  |  |  |  |
| **Maybridge database** |  |  |  |  |  |  |  |  |
| LIGAND10436 | 154.168 | 3 | 6 | -0.859 | -1.13 | -3.286 | 125.584 | 59.484 |
| LIGAND7497 | 166.197 | 1 | 4 | 0.825 | -2.516 | -3.899 | 634.788 | 81.939 |
| LIGAND6555 | 152.152 | 2 | 3.5 | 0.464 | -1.13 | -3.427 | 687.454 | 80.444 |
|  |  |  |  |  |  |  |  |  |
| **ZINC database** |  |  |  |  |  |  |  |  |
| ZINC18141652 | 194.141 | 4 | 9.8 | -2.03 | -0.376 | -1.262 | 3.574 | 24.959 |
| **ZINC03871163** | 196.218 | 3.8 | 8.3 | -1.308 | -0.389 | -2.866 | 89.098 | 54.183 |
| ZINC00901606 | 156.098 | 0 | 2.5 | -0.055 | -0.662 | -0.907 | 6.464 | 41.131 |
| **ZINC01583698** | 181.188 | 7 | 9.5 | -2.769 | 0.698 | -3.805 | 11.541 | 16.783 |
| ZINC08733367 | 125.13 | 1 | 2.5 | 0.538 | -1.075 | -3.179 | 572.481 | 79.456 |
| ZINC02597098 | 156.098 | 2 | 4 | -0.144 | -0.835 | 0.677 | 1.404 | 28.743 |
| ZINC02036492 | 174.196 | 1 | 4 | 1.228 | -1.746 | -1.534 | 74.793 | 67.672 |
