# Supplementary material for: Chemoinformatic Identification of Novel Inhibitors against Mycobacterium tuberculosis L-aspartate α-decarboxylase
Source: PLoS One. 2012 Mar 28;7(3):e33521. doi: 10.1371/journal.pone.0033521 (PMC3314653; doi:10.1371/journal.pone.0033521)
Supplement: Table S3 — Interactions of selected known inhibitors/ligands with MtbADC as verified by Glide XP. (DOCX) [file pone.0033521.s008.docx]

### Chemoinformatic identification of novel inhibitors against *Mycobacterium tuberculosis* L-aspartate α-decarboxylase

Reetu Sharma, Roopa Kothapalli, Antonius M.J. Van Dongen and Kunchithapadam Swaminathan

**Supplementary tables**

**Table S3**. Interactions of selected known inhibitors/ligands with MtbADC as verified by Glide XP.

| **Inhibitors/ligands** | **Glide score (kcal/mol)** | **Interacting residues** | | | | |
| --- | --- | --- | --- | --- | --- | --- |
| Phenylalanine | -2.72 | Pyr25 |  |  |  |  |
| β-hydroxy aspartate | -3.66 | Gly73 | Arg54 | ASN72 | PYR25 | TYR58 |
| L-cysteic acid | -2.66 | Thr57 | Pyr25 | Asn72 |  |  |
| Oxaloacetate | -4.96 | Tyr58 | Thr57 |  |  |  |
| D-serine | -4.20 | Gly73 | Pyr25 | Asn72 |  |  |
